# Crosstalk between Gross and Fine Motor Domains during Late Childhood: The Influence of Gross Motor Training on Fine Motor Performances in Primary School Children

**DOI:** 10.3390/ijerph182111387

**Published:** 2021-10-29

**Authors:** Vincenzo Sorgente, Erez James Cohen, Riccardo Bravi, Diego Minciacchi

**Affiliations:** Department of Experimental and Clinical Medicine, Physiological Sciences Section, University of Florence, 50134 Florence, Italy; vincenzo.sorgente@unifi.it (V.S.); erezjames.cohen@unifi.it (E.J.C.)

**Keywords:** motor development, gross motor skills, fine motor skills, physical education, physical activity

## Abstract

Gross and fine motor competence have a close relationship during development and are shown to correlate to some extent. However, the study of the interaction between these domains still requires further insights. In this study, we investigated the developmental changes in overall motor skills as well as the effects of gross motor training programs on fine motor skills in children (aged 6–11, *n* = 240). Fine motor skills were assessed before and after gross motor intervention using the Box and Block Test. The gross motor intervention was based on the Test of Gross Motor Development—3rd Edition. Results showed that gross and fine motor skills correlate across all years of primary school, both significantly improving with age. Finally, the gross motor intervention appeared to not influence fine motor skills. Our findings show that during primary school age, overall motor development is continuous, but non-linear. From age nine onward, there seems to be a major step-up in overall motor competence, of which teachers/educators should be aware of in order to design motor educational programs accordingly. While gross and fine motor domains might be functionally integrated to enhance children’s motor performances, further research is needed to clarify the effect of gross motor practice on fine motor performances.

## 1. Introduction

Motor skills refer to the underlying internal pathways responsible for moving the body through space as well as the cognitive processes that give rise to such movements [1]. These are classically divided into two categories, namely gross motor skills and fine motor skills [2]. Specifically, gross motor skills involve the body’s large muscles and pertain to movement of the trunk and limbs whereas fine motor skills involve the body’s small muscles and pertain to movements of wrists and fingers [3,4,5]. Moreover, gross motor skills are further categorized into locomotor and object control skills [6,7].

General development of motor skills undergoes major improvements during the formative years of childhood (i.e., 5–11 years of age) due to the maturation of the central and peripheral nervous system and locomotor system [8]. Research has shown that during child development, gross and fine motor competencies appear to have some correlation [9,10,11,12,13,14]. In fact, it was suggested that specific gross motor activities could involve fine motor adjustments (e.g., ball dribbling and handling, ball-striking with a bat, throwing at a target, skipping through a hopscotch-type pattern) [10,11]. Moreover, the same higher order neuromotor processes appear to be involved in the learning and mastering of both gross and fine motor skills [12]. Accordingly, gross and fine motor skills have been defined as motor domains that partially share the same cognitive processes [13].

Previous studies have investigated the relationship between gross and fine motor skills during various steps of children’s school education, obtaining contentious results when comparing gross and fine individual performance measures [9,13,14,15,16,17]. For instance, Cameron et al. [9] and Oberer et al. [13] showed moderate correlation between gross and fine motor skills. Specifically, Oberer et al. [13] reported a positive correlation in children aged 5.6–7.25 years, assessing both gross and fine motor skills using speed and precision tasks (e.g., jumping sideways and one leg stand for gross motor skills, posting coins, and drawing trail for fine motor skills). Similarly, Cameron and colleagues’ investigation [9] also reported a positive correlation in younger children (aged 3–4 years), assessing gross motor skills using balance, imitation, and hop and skip tasks, whereas fine motor skills were evaluated using spatial organization tasks (e.g., building a tower with bricks and drawing tasks). Furthermore, Dayem et al. [14] showed that an even higher correlation between gross and fine motor skills occurred in children aged 4–6 years, assessing gross motor skills using stationary, locomotor, and object manipulation tasks, while fine motor skills were assessed using a writing task. Conversely, other authors disagree with the positive correlation between gross and fine motor skills [15,16,17]. Specifically, Tortella and colleagues’ study [15] reported that there was no correlation between gross and fine motor skills in pre-school children aged 5–6 years, evaluating gross motor skills using precision, balance, throwing, and walking tasks, while fine motor skills were assessed using manual speed and precision tasks (e.g., building bricks and posting coins). Moreover, Souza et al. [16] found that when investigating global motor performance with the Bayley Scales of Infant and Toddler Development—Third Edition, there was a clear individual variability in overall motor proficiency as well as a weak correlation between gross and fine motor skills. Finally, Amaro et al. [17] reported no correlation between gross and fine motor skills in children aged 5–10 years when comparing the scores obtained in the “Körperkoordinationtest für kinder” and Minnesota manual dexterity test, respectively.

These contrasting results may be attributed to the fact that motor skills do not follow linear developmental trajectories [16,18]. Hence, it is not surprising that investigating children of different ages could produce different results. Furthermore, these studies assessed motor skills during short age spans using heterogeneous tasks. To our knowledge, a broader investigation regarding the gross-fine motor development during the entire late childhood developmental stage (i.e., primary school children) with consistent motor assessment methods has yet to be carried out.

Despite the mentioned elements of relationship during development, the influence of gross motor training on fine motor skill enhancement in school age children has not been adequately assessed in the literature. Indeed, research has mainly focused on interactions between gross-fine motor skills and other competence domains (e.g., social skills [19,20], cognitive skills [21,22], academic achievement [23,24]), indicating a positive influence of both gross and fine motor skills on these elements [20,25,26]. However, a specific approach aiming to explore the influence that components of gross and fine motor domains exert on each other is missing. Given that both gross and fine motor skills hold a mutual influence on these fundamental factors for children’s overall well-being, it is plausible that gross and fine motor skills share some elements, which the enhancement of one (i.e., gross motor skills) could also improve the other (i.e., fine motor skills).

In the current study, we sought to expand the focus pertaining to the motor development of primary school children in two directions: first, to investigate developmental changes in overall motor skills during late childhood, which was achieved by comparing both gross motor training results as well as fine motor performances among the different ages. Second, to examine the effects of short gross motor training programs on fine motor skills in children, which was achieved by comparing the pre- and post-training results for the evaluation of fine motor skills.

## 2. Materials and Methods

### 2.1. Participants

A total of 240 typically developing male and female children from age six to 11 participated in this study. All subjects were free of any documented visual, motor, and/or neurological impairments, nor any intellectual disabilities. None of the participants were involved in extracurricular sports practice. The study protocol was approved by the institution ethics committee, Prot. N.0018234E, Rif. 63/12. Prior to the start of the study, written informed consent was obtained from the parents/legal guardians of the children. Participants were all tested individually by the principal investigator and six research assistants, all of whom were familiar with the purpose of the study. The participants’ characteristics are summarized in Table 1.

In this study, fine motor skills were evaluated using the Box and Block Test (BBT) [27], whereas gross motor skill training sessions were conducted using the Test of Gross Motor Development—Third Edition (TGMD-3) [6]. Regarding the gross motor training, the total sample of subjects was divided into three subgroups: locomotor and ball skills subgroup (LBS), which executed all the TGMD-3 skills; locomotor skills subgroup (LS), which only executed the six LS subscale skills of TGMD-3; and the ball skills subgroup (BS), which only executed the seven BS subscale skills of TGMD-3 (Table 2). The purpose of the division was to observe whether the practice of a specific subset of skills could be more impactful on manual dexterity performance.

The reasons for dividing the sample into three groups were many, one being that gross motor skills are generally categorized into locomotor and object control [7]. Furthermore, the training of different types of gross motor skills have been shown to influence other aspects related to motor performance [28]. Moreover, short forms of the TGMD have already been conducted in the recent literature [29,30] for training/assessment of just one of the two subsets of gross motor skills included in the test (i.e., locomotor or object manipulation skills).

### 2.2. Procedures

The study spanned across five consecutive days, similar to previous studies [11,31] (Table 3). At the beginning of the study (i.e., day 1) each participant underwent a baseline evaluation for the fine motor skills of both the dominant and the non-dominant hand. Following that, participants took part in three gross motor skill training sessions (one session per day, i.e., days 2–4) which lasted from 30 to 45 min [32]. During these sessions, the participants’ gross motor skills were also evaluated. Finally, the same procedure for the evaluation of fine motor skills was also executed post-gross motor skills training (i.e., day 5). Both the fine motor skills evaluations and gross motor skills training sessions took place in indoor school gymnasiums.

### 2.3. Fine Motor Skills Evaluation

The BBT is a simple, validated, and suitable test that can be administered quickly to assess fine motor skills in children from age three onward [27,33,34]. The materials needed for the BBT are a wooden box (53.7 × 25.4 × 8.5 cm) divided into two compartments by a partition (15.2-cm-high) and 150 wooden cubic blocks (2.5 cm per side) [33,34]. We adopted the same procedure for both the fine motor skills evaluation of this study (i.e., baseline and post-gross motor skills training). Each subject was seated on a height-adjustable chair, with the forearms resting on a desk. Both hands were tested separately, starting with the dominant hand, which was determined by asking the participants to write their name on paper [27]. All 150 cubes were placed in one compartment. The test consisted in transferring as many blocks as possible, one block at a time and with one hand, from one compartment to the other in 60 s. Each test was preceded by a 15 s practice period. The cube placement always allows for lateral to medial movements (i.e., when testing the right hand, all the 150 cubes were placed in the right compartment of the box and had to be moved to the left compartment of the box). The number of blocks transferred in 60 s was the outcome score of the test. The maximum total score possible for a single trial was 150, meaning that in 60 s, all cubes were moved from the lateral compartment to the medial compartment.

### 2.4. Gross Motor Skills Training

The TGMD-3 is a direct observation assessment that measures the performance of various gross motor skills in children ages 3–10.9 years [35]. The continued popularity of the TGMD has been associated with its increasing use in research in child development, physical activity, and public health [36,37,38,39,40]. Particularly, the latest edition of the TGMD (i.e., the TGMD-3) has been proposed as a valid and reliable assessment tool for measuring gross motor skills competence in both pre-school and primary school children [41,42,43,44].

The skills present in the TGMD-3 include a selection of fundamental gross motor skills that are commonly taught in the primary physical education curriculum on an international scale [45]. Specifically, the TGMD-3 assesses 13 fundamental motor skills, partitioned into two subscales: locomotor skills and ball skills. The skills assessed in the locomotor subscale include run, gallop, one-legged hop, skip, jump, and slide. The skills assessed in the ball subscale include two-hand strike, one-hand strike, dribble, kick, catch, overhand throw, and underhand throw. Other than for motor skills assessment purposes, the TGMD has been suggested for training and improvement of specific motor skills [46].

In this study, testing stations were created for each skill (Table 4) and the evaluations were conducted observing the TGMD-3 assessment form guidelines [6], indicating the researcher to illustrate the proper execution of the skill, and then the subject to complete one practice trial, followed by two formal trials.

Each skill was evaluated by examining 3–5 performance criteria [45]. For instance, the gross motor skill named “dribbling” included three different criteria: make ball contact with one hand at the waist level; push (not slap) the ball with the fingertips; and maintain control for four consecutive bounces.

During the skill execution, the evaluator marked “1” in the score box for every performance criterion that the subject correctly demonstrated. If the subject did not demonstrate the appropriate criterion, a score of “0” was recorded in the score box. Total scores from the performance criteria over the two formal trials were summed to create a raw skill score. Raw skill scores were summed to provide a total raw score for either the locomotor or ball skill subscales or combined to provide a total TGMD-3 raw score. The maximum possible scores were 100 for the LBS subgroup, 46 for the LS subgroup, and 54 for the BS subgroup.

### 2.5. Data Collection

Data obtained consisted of the scores that subjects were given for the fine motor skills evaluations of day 1 (i.e., baseline) and day 5 (post gross motor training). Scores of the gross motor training were taken on day 2, day 3, and day 4. Although we collected data from gross motor training sessions, the TGMD-3 scores were only used for comparison between ages and groups, and not as a measure of gross motor proficiency.

### 2.6. Statistics

Data were analyzed using MATLAB_R2020b software (The MathWorks, Inc., Natick, MA, USA). Nonparametric analyses were conducted since the Shapiro-Wilk test revealed a non-normal distribution of data (*p* < 0.001). In order to observe whether motor performances would differ based on sex, we conducted a Mann-Whitney U-test for independent variables comparing girls’ and boys’ BBT scores as well as the girls’ and boys’ TGMD-3 scores. Moreover, to observe whether gross and fine motor performances improve with age, we used the Spearman’s rank correlation coefficient among the subjects’ grades and BBT scores as well as the subjects’ grades and TGMD-3 scores. For the same purpose, the Kruskal-Wallis test for independent variables was conducted using the BBT scores with grades. Similarly, potential differences in gross motor activity due to age were investigated by conducting the Kruskal-Wallis test using the TGMD-3 scores with grades. The Kruskal-Wallis test was followed by the Dunn-Bonferroni adjusted post-hoc test in the case of multiple comparisons. Furthermore, aiming to investigate whether higher fine motor skills performances are related to higher gross motor performances, we used the Spearman correlation among the subjects’ scores in the BBT and TGMD-3. In addition, in order to observe whether there is a difference in fine motor skills performances before and after a short intervention of gross motor training, we conducted a Friedman test for dependent variables between BBT scores at the baseline and post-gross motor skills training.

## 3. Results

### 3.1. Sex Differences in Motor Performance

Descriptive data confronting the males’ and females’ BBT and TGMD-3 scores are reported in Table 5 and Table 6, respectively. Furthermore, we explored the possibility that motor performances would differ based on sex when comparing the girls’ BBT scores with the boys’ BBT scores by conducting a Mann-Whitney U-test for independent variables. In a similar fashion, we also compared the girls’ TGMD-3 scores with the boys’ TGMD-3 scores. No significant differences were found in both the BBT and TGMD-3 scores between the boys’ and girls’ performances for all subgroups and across all sessions (Table 7 and Table 8).

### 3.2. Developmental Progression of Gross Motor Skills

TGMD-3 scores improved with age in all subgroups (LBS, LS, and BS) across all sessions, albeit with some exceptions (Table 9).

In order to investigate the relationship of gross motor skills with age, we compared the subjects’ grade with TGMD-3 scores using the Spearman’s rank correlation coefficient. Correlation between grades and TGMD-3 scores were found to be low to moderate and significant for all of the three gross motor training sessions, ranging from R = 0.33 to 0.59, *p* < 0.001, except for the LS subgroup in the 3rd session with *p* < 0.05 (Table 10).

Moreover, in order to evaluate possible differences in gross motor skills due to age, we compared the subjects’ TGMD-3 scores with grades by conducting the Kruskal-Wallis test for independent variables, followed by the Dunn-Bonferroni adjusted post-hoc test for multiple comparison. The analysis returned mixed results among the different sessions for LBS, LS, and BS subgroups, as significant differences were found between grades in all subgroups and sessions, except for the LS subgroup in the third session (Table 11).

### 3.3. Developmental Progression of Fine Motor Skills

BBT scores improved with age for the dominant and the non-dominant hand both for the baseline and the post-gross motor skills training assessments (Table 12).

In order to investigate the relationship of fine motor skills with age, we compared the subjects’ grade with BBT scores using the Spearman’s rank correlation coefficient. Specifically, the correlation between grade and BBT scores was found to be high and significant for the baseline assessment for both the dominant and non-dominant hand. The same was also found for the post-TGMD-3 assessment (Table 13).

Moreover, in order to evaluate possible differences in fine motor skills due to age, we compared the subjects’ BBT scores with grades by conducting the Kruskal-Wallis test for independent variables, followed by the Dunn-Bonferroni adjusted post-hoc test for multiple comparison. The analysis showed significant differences for the BBT scores among grades, both in the dominant hand and non-dominant hand (Table 14).

All comparisons were found to be statistically significant (both dominant and non-dominant hand with *p* < 0.001) except for the 3rd vs. 4th grade (*p* = 0.11 in the dominant hand, *p* = 0.30 in non-dominant hand) and the 4th vs. 5th grade (*p* = 0.10 in the dominant hand, *p* = 0.72 in non-dominant hand). As for the post TGMD-3 assessment, results were similar, as significant differences were found for the BBT scores among grades, both in the dominant hand and non-dominant hand. All comparisons were found to be statistically significant (both dominant and non-dominant hand with *p* < 0.001) except for the 3rd vs. 4th grade (*p* = 0.18 in the dominant hand, *p* = 0.27 in non-dominant hand) and the 4th vs. 5th grade (*p* = 0.17 in the dominant hand, *p* = 0.11 in the non-dominant hand).

### 3.4. Crosstalk between Gross and Fine Motor Skills

In order to investigate the relationship between fine gross and fine motor skills, the Spearman correlation was implemented on the subjects’ BBT and TGMD-3 scores (Table 15).

As for the LBS subgroup, results showed a statistically significant low to moderate correlation between the BBT and TGMD-3 scores (ranging from R = 0.48 to 0.62, *p* < 0.001). Regarding the LS subgroup, the correlation between the BBT and TGMD-3 scores were found to be low (ranging from R = 0.24 to 0.40), though statistically significant for all sessions (*p* < 0.05). Concerning the BS subgroup, the analysis indicated similar results to the LBS subgroup, thus a significant low to moderate correlation between the BBT and TGMD-3 scores (ranging from R = 0.40 to 0.57, *p* < 0.001).

**Figure 1:**
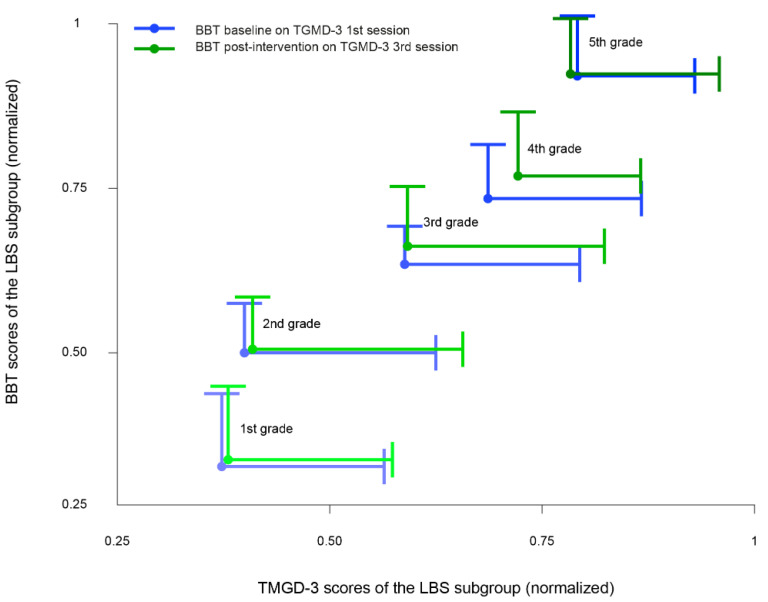
Results for gross and fine motor proficiency for the LBS subgroup. The scores are reported as a normalized mean of the BBT and the TGMD-3 per grade (dots) and standard deviations (lines). Performances gradually improved with age for both gross and fine motor skills, but this overall improvement was more evident between the 1st and 4th grade, 1st and 5th grade, 2nd and 4th grade, and 2nd and 5th grade, as evidenced by the significant difference (*p* < 0.001) in general motor proficiency (see Table 11 and Table 14). Conversely, no significant differences were found in both gross and fine motor performances as the sessions progressed (see Table 16 and Table 17).

Furthermore, both gross and fine motor skills seemed to improve as sessions progressed, with the exception of the 5th grade (Figure 1).

In order to investigate potential differences in gross motor skills due to the training intervention, the Friedman test was implemented on the TGMD-3 scores for all sessions (i.e., between the 1st and 2nd session, the 2nd and 3rd session, and the 1st and 3rd session). Though TGMD-3 scores seemed to improve as the sessions progressed (Figure 1), none of the changes among the sessions of gross motor training were found to be statistically significant (Table 16).

In order to investigate potential differences in fine motor performance due to gross motor training intervention, the Friedman test was implemented on the BBT scores before and after the TGMD-3 sessions (i.e., baseline and post-TGMD-3 training). Though BBT scores seemed to generally improve from baseline to post-TGMD-3 intervention (Figure 1), no significant results were found for both the dominant hand and non-dominant hand (Table 17).

## 4. Discussion

The purpose of the present study was to expand the focus pertaining to motor development during primary school age, at the same time investigating the influence of different short gross motor training on fine motor performance. The results showed a general improvement for both gross and fine motor performances with age (Table 9 and Table 12). Moreover, our findings confirm previous observations [9,13] showing a moderate to high correlation between gross and fine motor skills (Table 15). However, neither the improvements nor declines in a single grade performances were found to be significant (*p* > 0.05) throughout the experiment for both gross (Table 16) and fine motor performances (Table 17). Regarding the gross motor intervention, it is worth mentioning that we used the same test battery to train and assess the exact same skills during the experiment, which could result in a training effect of the specific skills. However, as the scope of the gross motor measurement was to investigate developmental changes (i.e., older children tend to perform better than younger ones with a continuous but non-linear trend), our experimental design did not intend to evaluate changes in gross motor performance across the intervention. Furthermore, it was possible to observe some differences in the developmental path among the three subgroups of gross motor training. Specifically, the LBS and BS subgroup presented consistent differences (*p* < 0.001) between children aged 6–8 and 9–11, while the LS subgroup presented less consistent differences over gross motor sessions between children aged 6–7 and 10–11 (Table 11). Concerning the influence of gross motor practice on fine motor skills, no differences in fine motor performances were found following the gross motor practice program (Table 17). Regarding sex-related motor performance, no differences were found between male and female performances for both gross and fine motor skills (Table 7 and Table 8).

In this study, both gross and fine motor skills significantly improved with age (Table 11 and Table 14). This trend was expected as competency in fundamental movement skills has been shown to follow an increasing developmental trajectory, with both gross and fine motor skills improving with chronological age [47,48,49]. Moreover, Bolger et al. [50] already observed that in primary school children, older children scored significantly higher than their younger peers in both locomotor and object-control scores in the TGMD-2. Therefore, in relation to the current literature and our findings, it seems that during primary school years, children’s gross and fine motor competence continuously improve with age (see Table 11 and Table 14 and Figure 1).

As was observed, as gross and fine motor skills follow different developmental paths [16,51], it is not surprising that we found the age-fine motor skills correlation to be higher than the age-gross motor skills correlation (Table 10 and Table 13). Moreover, fine motor performances did not differ between the 3rd and 4th grade and between the 4th and 5th grade (i.e., between children aged 9–10 and aged 10–11), although significant differences were found between the 3rd and 5th grade (i.e., between children aged 9 and 11, see Table 14). A possible explanation for this trend is that children aged 9–11 experience a period of stabilization in the physical growth and consolidation of both cognitive and neuro-motor abilities [52,53,54,55,56,57,58]. However, it should be mentioned that while for fine motor skills evaluation we used a single task test (i.e., the BBT), for gross motor assessment, we used a test battery of diversified tasks (i.e., the TGMD-3). As the scope of this paper was to provide a general evaluation of the effect of different types of gross motor training on fine motor performance, we limited the fine motor skills evaluation to a single test (widely used in the literature [27,32,33,34]). As gross motor control is generally considered as a sum of different subsets of skills [7], it is fitting to use different tasks when designing an overall gross motor training intervention. On the other hand, fine motor control is not classically defined by different subsets of skills, and was used in this study as an output measure. Furthermore, other studies have already compared a different number of tasks between gross and fine motor training tests [9,13,14,15,17,30]. Nonetheless, it is possible that the lower correlation and heterogeneity of results we found in the gross motor assessment among grades could be due to the higher request of motor variability [59]. Certainly, a different combination of gross and fine motor skills assessments could provide different results. However, this would go beyond the scope of this paper. Future studies should clarify the aspects related to the influence of different combination of gross and fine motor skills assessments on overall motor performance.

As for the relationship between gross and fine motor skills, high scores on gross motor skills assessment reflected high scores in fine motor skills evaluation across all the sessions of this study and for all the gross motor activity subgroups. These results are in line with the research work of Oberer et al. [13], which suggested a moderate level of relationship between gross and fine motor skills. In addition, Cameron et al. [9] already indicated that children with higher scores in fine motor skills evaluations tended to score higher on the gross motor assessments compared to children with lower scores in gross or fine motor assessments. However, it is worth noting that while both studies were conducted on kindergarten children [9,13], this study expanded the scope of the gross-fine motor relationship to the primary school years. Therefore, including the primary educational stage to the topic of gross–fine motor relationship, this study contributed to further explore the features of overall motor development across all years of childhood.

Although we found a correlation between gross and fine motor skills, this cannot be attributed to a direct influence of the gross motor training used in this study on fine motor performance. This apparent lack of influence may suggest that the designed gross motor training intervention was not adequate for influencing fine motor skills in the short-term, regardless of the type of gross motor activity. The intervention duration might be a critical point (i.e., three sessions of gross motor practice to influence fine motor performance). In this regard, although some studies have also shown that a short-term intervention could influence motor performances [30,60], the general notion is that short-term intervention would not elicit great changes in performances [61,62]. Thus, it is not surprising that the gross motor training in this study did not bring significant differences in fine motor performance. The results support the notion of a certain critical point for intervention duration [61,62]. Hence, more training time may be needed to observe a significant influence of gross motor training on fine motor skills.

Another doubtful element could be related to the design of the gross motor training program. Seeing that in this study we used three different motor training programs composed of numerous tasks (i.e., the TGMD-3 locomotor skills battery, ball skills battery, and the complete battery), it might be appropriate to focus on a reduced number of tasks for efficiently stimulating gross motor skills. As previously mentioned, this would reduce the demand for motor variability, allowing subjects to perform a more specific training that would return less heterogeneous results than those present in this study. Though the training used in this study was relatively task specific, it has been widely used in the literature for the assessment of general gross motor proficiency [35,36,37,38,39,40,41,42,43,44,45]. However, future studies will benefit from the examination of developmentally focused training as well as neutral training programs. Moreover, having found no differences in the type of gross motor training carried out, we would suggest future research to keep investigating the influence of both locomotor and object manipulation training on fine motor performance.

The topic of motor skills differences between male and female children appears to be contentious in the research literature. As for this study, no differences were found between male and female motor performances (Table 7 and Table 8). These results are in line with various research work [57,63] that suggested that there are no sex differences in fine motor skills in primary school children. In addition, Slykerman et al. [64] indicated that there may be no sex differences in the locomotor skills in children with a mean age of 6.5 years. However, other studies suggest otherwise. For instance, when assessing gross motor skills in school age children, Bolger et al. [50] found that boys scored significantly higher than girls in the object-control score while girls scored significantly higher in the locomotor score. For ages 7–8, it has been noted that boys develop ball skills earlier than girls and that girls acquire fine motor skills before boys [65]. Based on the results of this study and on recent literature, our cautious position is that when considering the whole late childhood development stage, there may be no clear differentiation between males and females in overall motor performances. However, these aspects should be investigated with further and dedicated research.

## 5. Conclusions

The study of interactions between gross and fine motor skills is important for identifying novel strategies to enhance motor learning during childhood. This research expands current findings regarding the relationship between gross and fine motor skills to the late childhood developmental stage. Moreover, our results align with previous findings regarding the positive correlation between gross and fine motor skills. Although we did not observe a short-term influence of gross motor practice on fine motor control, it is possible that longer interventions could provide a more prominent effect on fine motor performances. Thus, designing other types of interventions could be useful to deepen the interaction between the gross and fine motor domains during the children’s school years. Finally, this study showed that overall motor development appears to follow a specific trajectory in primary school subjects. In particular, both males and females aged 9–11 seem to experience a major step-up in both gross and fine motor proficiency compared to their younger peers (ages 6–8). Other than an academic audience, these findings are also valuable for teachers, educators, and trainers (i.e., professionals who design and put into practice children’s motor educational programs). Further studies are needed to better clarify the relationship between gross and fine motor proficiency during different stages of primary school education.

## Figures and Tables

**Table 1:** Characteristics of the participants.

Grade	Age (Years)	*n*	Boys	Girls	Age (Years)	
1st Grade	6–7	66	40	26	M ^1^ ± SD ^2^	8.57 ± 2.33
2nd Grade	7–8	50	29	21	Range	6–10.6
3rd Grade	8–9	48	30	18		
4th Grade	9–10	45	27	18		
5th Grade	10–11	31	20	11		
Total		240	127	113		

^1^ M = mean; ^2^ SD = standard deviation.

**Table 2:** Number of subjects per grade and subgroups of gross motor skills training.

Grade	LBS ^1^	LS ^2^	BS ^3^
1st Grade	22	22	22
2nd Grade	17	16	17
3rd Grade	17	16	15
4th Grade	15	15	15
5th Grade	11	9	11
Total (%)	77 (32.1%)	81 (33.7%)	82 (34.1%)

^1^ LBS = Locomotor and ball skills subgroup. ^2^ LS = Locomotor skills subgroup. ^3^ BS = Ball skills subgroup.

**Table 3:** Timeline of the study.

Activity	FMS ^1^ Evaluation (Baseline)	GMS ^2^ Training			FMS ^1^ Evaluation (Post GMS ^2^ Training)
(Test)	BBT ^3^	TGMD-3 ^4^			BBT ^3^
Day #	Day 1	Day 2	Day 3	Day 4	Day 5
Test time	5–10 min	30–45 min			5–10 min

^1^ FMS = Fine Motor Skills; ^2^ GMS = Gross Motor Skills; ^3^ BBT = Box and Block Test; ^4^ TGMD-3 = Test of Gross Motor Development—Third Edition.

**Table 4:** Equipment needed for the TGMD-3 stations.

Locomotor Skills	Equipment	Material	Measures
ALL	Mini markers	Polyethylene	Base diameter 9.52 cm, height 16.51 cm
Ball Skills	Equipment	Material	Measures
Two hand strike	Batting tee Baseball Bat	Rubber, latex free Rigid polyethylene Plastic	44.19 cm 43.69 cm 7.62 cm Diameter 7.62 cm Barrel diameter 5.72 cm, height 76.2 cm
One hand strike	Pickleball paddle Tennis ball	Plastic Rubber and latex	Length 35.6 cm, plastic grid 1.3 cm Non-pressurized
Dribbling	Playground balls	Nylon and rubber	Diameter 21.59 cm
Kicking	Playground balls	Nylon and rubber	Diameter 21.59 cm
Catch	Baseball	Rigid polyethylene	Diameter 7.62 cm
Overhand throw	Tennis ball	Rubber and latex	Non-pressurized
Underhand throw	Tennis ball	Rubber and latex	Non-pressurized

**Table 5:** Scores ^1^ of the BBT divided by sex (males M, females F).

Dominant Hand				Non-Dominant Hand			
Baseline		Post		Baseline		Post	
M	F	M	F	M	F	M	F
55 (7)	54 (7)	50.5 (9)	50 (8)	55 (8)	54 (7)	51 (9.25)	50 (8)

^1^ Median (interquartile range).

**Table 6:** Scores ^1^ of the TGMD-3 divided by sex (males M, females F).

LBS ^2^ Subgroup						LS ^3^ Subgroup						BS ^4^ Subgroup					
Sessions						Sessions						Sessions					
1st		2nd		3rd		1st		2nd		3rd		1st		2nd		3rd	
M	F	M	F	M	F	M	F	M	F	M	F	M	F	M	F	M	F
80 (13)	81 (14)	82 (15)	81 (16)	85 (13)	82 (13)	46 (9)	44 (9)	45 (7)	42 (8.5)	46 (8)	44 (10)	38 (7.3)	36 (5)	38 (5.3)	36 (8.5)	38 (5)	37 (5.5)

^1^ Median (interquartile range), ^2^ LBS = Locomotor and ball skills subgroup, ^3^ LS = Locomotor skills subgroup, ^4^ BS = Ball skills subgroup.

**Table 7:** Mann-Whitney U-test between male and female BBT scores.

	Data	Baseline	Post
Dominant hand	U	7025	7188.5
	Z	0.31	0.62
	*p*	0.76	0.53
Non-dominant hand	U	7181	7591.5
	Z	0.61	1.40
	*p*	0.54	0.16

**Table 8:** Mann-Whitney U-test between male and female TGMD-3 scores.

Subgroup	Data	1st Session	2nd Session	3rd Session
LBS ^1^	U	762.5	748.5	724.5
	Z	0.43	0.56	0.79
	*p*	0.67	0.58	0.43
LS ^2^	U	618	582.5	670
	Z	1.38	1.74	0.86
	*p*	0.17	0.84	0.39
BS ^3^	U	529.5	526.5	517
	Z	1.63	1.67	1.76
	*p*	0.10	0.94	0.08

^1^ LBS = Locomotor and ball skills subgroup. ^2^ LS = Locomotor skills subgroup. ^3^ BS = Ball skills subgroup.

**Table 9:** TGMD-3 scores ^1^ for all grades and all subgroups of gross motor skills training.

	LBS ^2^ Subgroup				LS ^3^ Subgroup			BS ^4^ Subgroup			
	Sessions				Sessions			Sessions			
Grade	1st	2nd	3rd	Grade	1st	2nd	3rd	Grade	1st	2nd	3rd
1st grade	74.5 (8.5)	75.5 (10)	78 (12.25)	1st grade	33 (6.75)	34.5 (7.5)	36 (3.25)	1st grade	40.5 (8.5)	39.5 (8.25)	40 (7.5)
2nd grade	75 (12.5)	75 (16)	77 (15.5)	2nd grade	36 (4.25)	37.5 (8.75)	37 (6.75)	2nd grade	45 (11.5)	45 (9)	43 (10)
3rd grade	82 (11.5)	82 (12.5)	84 (11.5)	3rd grade	37 (3.75)	38 (4.75)	37.5 (5.75)	3rd grade	45 (8)	44 (7)	45 (6)
4th grade	87 (11)	86 (9)	87 (9)	4th grade	38 (8)	40 (5)	39 (7)	4th grade	47 (6)	45 (3)	48 (6)
5th grade	87 (9)	90 (9)	91 (10)	5th grade	39 (5)	40 (5)	38 (3.5)	5th grade	51 (5)	51 (3)	50 (2)

^1^ Median (interquartile range), ^2^ LBS = Locomotor and ball skills subgroup, ^3^ LS = Locomotor skills subgroup, ^4^ BS = Ball skills subgroup.

**Table 10:** Spearman’s rank correlation coefficient between TGMD-3 scores and grades.

Subgroup	1st Session	2nd Session	3rd Session
LBS ^1^	R = 0.52,	R = 0.59,	R = 0.53,
	*p* < 0.001	*p* < 0.001	*p* < 0.001
LS ^2^	R = 0.39,	R = 0.42,	R = 0.33,
	*p* < 0.001	*p* < 0.001	*p* < 0.001
BS ^3^	R = 0.55,	R = 0.50,	R = 0.54,
	*p* < 0.001	*p* < 0.001	*p* < 0.001

^1^ LBS = Locomotor and ball skills subgroup, ^2^ LS = Locomotor skills subgroup, ^3^ BS = Ball skills subgroup.

**Table 11:** Kruskal-Wallis test followed by the Dunn-Bonferroni post-hoc between TGMD-3 scores and grades.

Subgroup	Data	1st Session	2nd Session	3rd Session
LBS ^1^	Chi Sq	33.53	29.84	24.00
	d.f. ^4^	4	4	4
	*p*	<0.001	<0.001	<0.001
	Post-hoc ^5^	1 vs.4; 1 vs.5; 2 vs.4; 2 vs.5	1 vs.4; 1 vs.5; 2 vs.4; 2 vs.5	1 vs.4; 1 vs.5; 2 vs.4; 2 vs.5
LS ^2^	Chi Sq	13.41	13.53	8.94
	d.f. ^4^	4	4	4
	*p*	<0.05	<0.05	=0.06
	Post-hoc ^5^	1 vs.5	1 vs.4; 1 vs.5	//
BS ^3^	Chi Sq	26.8	27.89	26.69
	d.f. ^4^	4	4	4
	*p*	<0.001	<0.001	<0.001
	Post-hoc ^5^	1 vs.4; 1 vs.5; 2 vs.5	1 vs.5; 2 vs.5; 3 vs.5; 4 vs.5	1 vs.4; 1 vs.5; 2 vs.5

^1^ LBS = Locomotor and ball skills subgroup. ^2^ LS = Locomotor skills subgroup. ^3^ BS = Ball skills subgroup. ^4^ d.f. = degrees of freedom. ^5^ Refers to the significant difference (*p* < 0.05) between the single grades.

**Table 12:** BBT scores^1^ comparison between the baseline and post-gross motor skills training assessments.

	Dominant Hand		Non-Dominant Hand	
Grade	Baseline	Post	Baseline	Post
1st grade	50 (3)	52 (2)	45 (2)	46 (1)
2nd grade	53 (2)	54 (2)	49.5 (2.25)	50 (2.25)
3rd grade	56 (1.75)	57 (2.25)	53.5 (3)	54 (2)
4th grade	57 (2)	59 (3)	54 (3)	55 (2)
5th grade	62 (3)	63 (4)	56 (3)	57 (3)

^1^ Median (interquartile range).

**Table 13:** Spearman’s rank correlation coefficient between BBT scores and grades.

Dominant Hand		Non-Dominant Hand	
Baseline	Post	Baseline	Post
R = 0.95, *p* < 0.001	R = 0.94, *p* < 0.001	R = 0.94, *p* < 0.001	R = 0.92, *p* < 0.001

**Table 14:** Kruskal-Wallis test followed by the Dunn-Bonferroni post-hoc between BBT scores and grades.

	Data	Baseline	Post
Dominant hand	Chi Sq	206.8	203.48
	d.f. ^1^	4	4
	*p*	<0.001	<0.001
	Post-hoc exceptions ^2^	3 vs.4; 4 vs.5	3 vs.4; 4 vs.5
Non-dominant hand	Chi Sq	203.48	200.48
	d.f. ^1^	4	4
	*p*	<0.001	<0.001
	Post-hoc exceptions ^2^	3 vs.4; 4 vs.5	3 vs.4; 4 vs.5

^1^ d.f. = degrees of freedom. ^2^ Refers to the non-significant differences (*p* > 0.05) between single grades.

**Table 15:** Spearman correlation between BBT scores and TGMD-3 scores.

LBS ^1^				
BBT Evaluations		1st Session	2nd Session	3rd Session
Baseline	Dominant hand	R = 0.56 *p* < 0.001	R = 0.56 *p* < 0.001	R = 0.50 *p* < 0.001
	Non-dominant hand	R = 0.58 *p* < 0.001	R = 0.57 *p* < 0.001	R = 0.53 *p* < 0.001
Post	Dominant hand	R = 0.57 *p* < 0.001	R = 0.55 *p* < 0.001	R = 0.48 *p* < 0.001
	Non-dominant hand	R = 0.62 *p* < 0.001	R = 0.61 *p* < 0.001	R = 0.56 *p* < 0.001
LS ^2^				
Baseline	Dominant hand	R = 0.39 *p* < 0.001	R = 0.40 *p* < 0.001	R = 0.33 *p* < 0.05
	Non-dominant hand	R = 0.33 *p* < 0.05	R = 0.38 *p* < 0.001	R = 0.24 *p* < 0.05
Post	Dominant hand	R = 0.40 *p* < 0.001	R = 0.43 *p* < 0.001	R = 0.36 *p* < 0.05
	Non-dominant hand	R = 0.36 *p* < 0.05	R=0.40 *p* < 0.001	R = 0.27 *p* < 0.05
BS ^3^				
Baseline	Dominant hand	R = 0.55 *p* < 0.001	R = 0.51 *p* < 0.001	R = 0.57 *p* < 0.001
	Non-dominant hand	R = 0.54 *p* < 0.001	R = 0.47 *p* < 0.001	R = 0.50 *p* < 0.001
Post	Dominant hand	R = 0.56 *p* < 0.001	R = 0.51 *p* < 0.001	R = 0.55 *p* < 0.001
	Non-dominant hand	R = 0.48 *p* < 0.001	R = 0.40 *p* < 0.001	R = 0.45 *p* < 0.001

^1^ LBS = Locomotor and ball skills subgroup. ^2^ LS = Locomotor skills subgroup. ^3^ BS = Ball skills subgroup.

**Table 16:** Statistical significance (*p*-value) of the changes in gross motor performance during the intervention.

	LBS ^1^ Subgroup				LS ^2^ Subgroup			BS ^3^ Subgroup			
	Sessions				Sessions			Sessions			
Grade	1st vs. 2nd	2nd vs. 3rd	1st vs. 3rd	Grade	1st vs. 2nd	2nd vs. 3rd	1st vs. 3rd	Grade	1st vs. 2nd	2nd vs. 3rd	1st vs. 3rd
1st grade	*p* = 0.16	*p* = 0.82	*p* = 0.06	1st grade	*p* = 0.08	*p* = 0.13	*p* = 0.65	1st grade	*p* = 0.52	*p* = 0.28	*p* = 0.15
2nd grade	*p* = 0.24	*p* = 0.25	*p* = 0.36	2nd grade	*p* = 0.19	*p* = 0.91	*p* = 0.31	2nd grade	*p* = 0.38	*p* = 0.77	*p* = 0.82
3rd grade	*p* = 0.31	*p* = 0.69	*p* = 0.45	3rd grade	*p* = 0.11	*p* = 0.72	*p* = 0.59	3rd grade	*p* = 0.26	*p* = 0.54	*p* = 0.69
4th grade	*p* = 0.22	*p* = 0.18	*p* = 0.34	4th grade	*p* = 0.85	*p* = 0.45	*p* = 0.53	4th grade	*p* = 0.96	*p* = 0.39	*p* = 0.14
5th grade	*p* = 0.09	*p* = 0.45	*p* = 0.21	5th grade	*p* = 0.68	*p* = 0.78	*p* = 0.56	5th grade	*p* = 0.17	*p* = 0.16	*p* = 0.33

^1^ LBS = Locomotor and ball skills subgroup, ^2^ LS = Locomotor skills subgroup, ^3^ BS = Ball skills subgroup.

**Table 17:** Friedman’s test between BBT scores at the baseline and BBT scores post gross motor skills training.

	Dominant Hand			Non-Dominant Hand		
Grade	*p*-Value	Chi-Sq	d.f. ^1^	*p*-Value	Chi-Sq	d.f.
1st grade	0.43	0.68	1	0.74	0.63	1
2nd grade	0.11	0.50	1	0.88	0.41	1
3rd grade	0.24	0.86	1	0.66	0.74	1
4th grade	0.09	0.25	1	0.13	0.13	1
5th grade	0.08	0.19	1	0.25	0.36	1

^1^ d.f. = degrees of freedom.

## Data Availability

Data are available on request.
